# Real-time on-device weed identification using a hardware-efficient lightweight CNN

**DOI:** 10.3389/fpls.2026.1747863

**Published:** 2026-02-16

**Authors:** Yuxuan Zhang, Yuchen Lu, Luciano Sebastian Martinez-Rau, Quan Qiu, Sebastian Bader

**Affiliations:** 1 College of Intelligent Science and Engineering, Beijing University of Agriculture, Beijing, China; 2 Department of Computer and Electrical Engineering, Mid Sweden University, Sundsvall, Sweden; 3 Yantai Research Institute, Harbin Engineering University, Yantai, China; 4 Instituto de Investigación en Señales, Sistemas e Inteligencia Computacional, sinc(i), FICH-UNL/CONICET, Santa Fe, Argentina

**Keywords:** embedded systems, energy-efficient computing, lightweight convolutional neural network (CNN), on-device inference, precision agriculture, TinyML, weed identification

## Abstract

Accurate and timely weed identification is fundamental to sustainable crop management, particularly for autonomous agricultural systems operating under strict energy and hardware constraints. While deep learning has significantly advanced image-based weed recognition, most existing models rely on GPU-based inference and therefore cannot be deployed directly in low-power field devices. In this study, we propose a hardware-efficient lightweight convolutional neural network (CNN), named TinyWeedNet, designed specifically for real-time on-device weed identification in precision agriculture. The model integrates multi-scale feature extraction, depthwise separable inverted residual blocks, and compact channel attention to enhance discriminative ability while maintaining a minimal computational footprint. To evaluate its suitability for field deployment, TinyWeedNet was trained and tested on the public DeepWeeds dataset and implemented on an STM32H7 microcontroller via the TinyML workflow. Experimental results demonstrate that the model achieves 97.26% classification accuracy with only 0.48 M parameters, supporting sub-90 ms inference and low energy consumption during fully embedded execution. A comprehensive analysis, including benchmark comparisons, hyperparameter sensitivity tests, and ablation studies, demonstrates that TinyWeedNet provides a good balance of accuracy, speed, and energy efficiency for resource-constrained agricultural platforms. Overall, this work demonstrates a practical pathway for integrating real-time, low-power weed identification into field robots, UAVs, and distributed sensing nodes, contributing to more autonomous and energy-aware weed management strategies in precision agriculture.

## 1 Introduction

Agricultural weed infestation poses a persistent threat to global crop production, competing with crops for nutrients, water, and sunlight while increasing reliance on herbicides Dadashzadeh et al. (2024); Sathesh et al. (2025b); Jiménez et al. (2024); Sathesh and Maheswaran (2024). Effective weed management is therefore essential for improving yield stability and supporting sustainable agricultural practices Sathesh et al. (2025a); Parvis and Pirola (1999); Anand et al. (2021); Holmes et al. (2019). Traditional methods, such as uniform herbicide spraying and manual removal, are increasingly unsuitable due to ecological risks, labor intensity, and rising operational costs. Consequently, real-time and accurate weed identification has become a central requirement for autonomous field robots, UAVs, and smart farming systems that aim to implement precise and environmentally responsible weed control Lottes et al. (2017); Wu et al. (2020).

Recent advances in agricultural computer vision have leveraged deep learning (DL) techniques to achieve high weed–crop discrimination accuracy across diverse field conditions Sathesh et al. (2024); Upadhyay et al. (2025); Luo et al. (2023); Ishak et al. (2009); Czymmek et al. (2019); Hussmann et al. (2019); Maheswaran et al. (2024). High-capacity neural models such as ResNet, Inception, and transformer-based architectures perform well on benchmark datasets including DeepWeeds Olsen et al. (2019). For example, Sunil et al. employed VGG16 to classify four weed species (crabgrass, kochia, ragweed, and waterhemp) and six crop species (black bean, rapeseed, maize, flax, soybean, and sugar beet), achieving an accuracy of 93–97.5% Sunil et al. (2022). Liu et al. achieved a spraying completion rate of 93% for target weeds, including Senecio vulgaris and Capparis spinosa, in strawberry fields, thereby reducing pesticide use and associated environmental risks Liu et al. (2021). Garibaldi et al. reported that the Xception model achieved accuracies ranging from 83% to 97% for early-stage weed classification in maize fields under natural illumination conditions Garibaldi-Márquez et al. (2022). However, their heavy computational and memory demands make them impractical for low-power devices that operate autonomously in the field. While lightweight CNNs (such as MobileNet, SqueezeNet, and ShuffleNet Sandler et al. (2018); Iandola et al. (2016); Tan and Le (2019)) reduce model complexity, most still exceed the strict limitations of microcontroller units (MCUs), which typically offer only a few hundred kilobytes of RAM and a limited energy budget. As a result, many current agricultural DL systems rely on cloud-based or offline processing Zhang et al. (2025c); Wiafe et al. (2025), limiting their ability to make immediate in-field decisions.

Tiny Machine Learning (TinyML) has recently emerged as a promising paradigm for bringing deep neural inference directly to small, energy-constrained devices Zhang (2025); Zhang et al. (2023, Zhang et al., 2025b). For example, Alshuhail et al. proposed a TinyML-based structural anomaly detection framework, achieving an anomaly detection accuracy of up to 92% while reducing energy consumption by 40% Alshuhail et al. (2025). Zeynali et al. applied TinyML to non-invasive blood glucose monitoring based on PPG signals; their method achieved 76.6% accuracy in Zone A and 23.4% in Zone B of the Clarke Error Grid Analysis (CEGA), indicating 100% clinical acceptability Zeynali et al. (2025). Asante et al. introduced TinyEEGConformer, which achieved an average accuracy of 79.63% on the BCI Competition IV 2a dataset, outperforming the baseline EEG Conformer model by 0.97% while reducing the number of parameters by a factor of 21 Asante et al. (2025). By executing models entirely on MCUs, TinyML enables low-latency, always-available, and energy-efficient weed detection without depending on external computation or network connectivity Zhang et al. (2025a). However, deploying CNNs in such constrained environments remains challenging. The model must provide reliable recognition of weeds with diverse morphological traits and under varying illumination, while simultaneously adhering to strict limits on memory footprint, computational cost, and energy consumption. Achieving this balance requires hardware-aware architectural designs rather than directly scaling down conventional CNNs.

To address these limitations, we propose TinyWeedNet, a hardware-efficient lightweight CNN tailored for real-time on-device weed identification on STM32-class MCUs. The model integrates multi-scale feature extraction, depthwise separable inverted residual blocks, and compact channel attention to enhance discriminative capability while minimizing computational demands. In addition to model development, we also conducted a comprehensive evaluation of benchmark comparisons, hyperparameter sensitivity analysis, and ablation studies to systematically assess how architectural parameters affect accuracy, latency, and power consumption during embedded execution.

The major contributions of this work are as follows:

- Hardware-efficient Lightweight Architecture: We design TinyWeedNet, a multi-scale, attention-enhanced lightweight CNN optimized for deployment on low-power MCUs commonly used in field robotics and embedded agricultural systems.
- Comprehensive Evaluation for Embedded Environments: We quantitatively analyze the trade-offs among accuracy, latency, memory footprint, and energy consumption through benchmark comparisons, hyperparameter sensitivity experiments, and ablation studies.
- Real-world Embedded Validation: We implement TinyWeedNet on an STM32H7 microcontroller, achieving 97.26% accuracy on DeepWeeds with sub-90 ms inference time and approximately 39 mJ energy per inference, demonstrating its suitability for real-time field deployment.

Together, these contributions provide a practical and validated pathway for bringing real-time weed identification to resource-constrained agricultural platforms, offering new opportunities for intelligent, energy-aware, and autonomous weed management in precision agriculture.

The rest of the paper is structured as follows. Section 2 describes the datasets used in this study, the preprocessing procedures, the proposed TinyWeedNet architecture, the experimental design, the MCU deployment toolchain, as well as the configurations for hyperparameter analysis, ablation studies and robustness studies. Section 3 presents the comparative experimental results, the hyperparameter sensitivity analysis, the ablation study, and the robustness analysis outcomes. Finally, Section 4 concludes the paper and outlines directions for future work.

## 2 Methodology

### 2.1 Dataset and preprocessing

The public DeepWeeds dataset comprises 17,509 RGB images representing eight Australian weed species along with a negative class consisting of non-target plants and background vegetation Olsen et al. (2019). These images were collected from eight distinct regions across northern Australia between June 2017 and March 2018. Classification on this species is particularly challenging due to the geographic and seasonal variability of the plants, the complexity of background environments, and the wide dynamic range of illumination conditions.

The eight weed species are Chinee apple (*Ziziphus mauritiana*), Lantana (*Lantana camara*), Parkinsonia (*Parkinsonia aculeata*), Sida (*Sida acuta*), Rubber vine (*Cryptostegia grandiflora*), Prickly acacia (*Vachellia nilotica*), Parthenium (*Parthenium hysterophorus*), and Snake weed (*Stachytarpheta jamaicensis*). The high inter-class similarity and significant intra-class variability further complicate the classification task. All images were captured using a FLIR Blackfly 23S6C camera and uniformly resized to 256 × 256 pixels in the original dataset.

**Figure 1:**
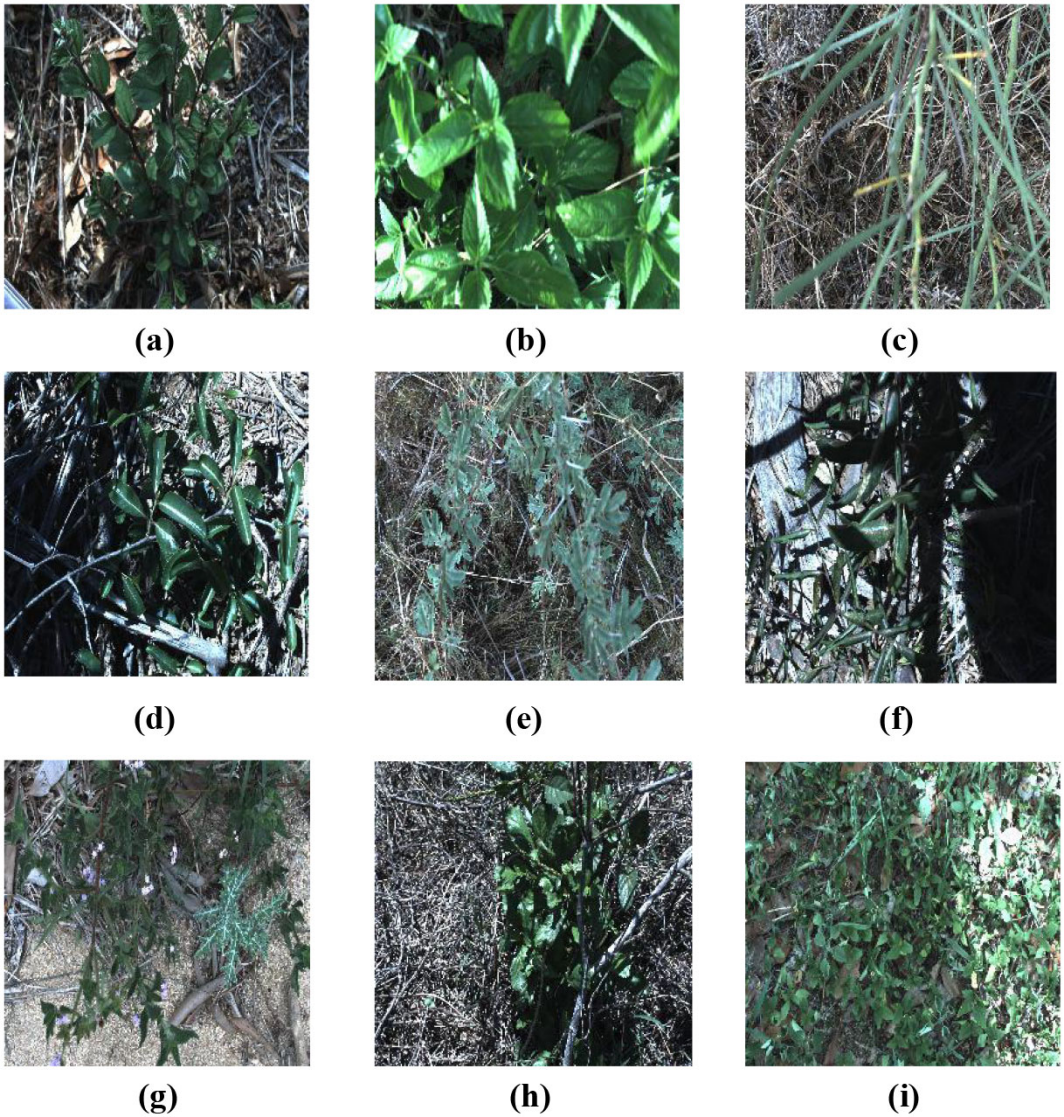
Weed image samples in the Deepweeds dataset **(a)** Chinese apple, **(b)** lantana, **(c)** parkinsonia, **(d)** parthenium, **(e)** prickly acacia, **(f)** rubber vine, **(g)** siam weed, **(h)** snake weed, and **(i)** negative class (non-target plants and background).

For this study, all images were resized to a uniform resolution of 224 × 224 × 3 pixels using bilinear interpolation in order to match the input specification of the proposed TinyWeedNet model. This resolution was selected as a compromise between preserving sufficient visual details for weed discrimination and maintaining computational efficiency suitable for MCU deployment. Pixel intensity values were converted from 8-bit integer format to floating-point and normalized to the range [0, 1] by dividing by 255. No additional color space transformation, histogram equalization, or manual filtering was applied, so as to preserve the original visual characteristics of the field-acquired images. The dataset was split into training and testing subsets following an 8:2 ratio using stratified sampling, ensuring that samples from all weed species were proportionally represented in both sets. Representative examples of the weed species from the DeepWeeds dataset are shown in **Figure 1**.

### 2.2 TinyWeedNet architecture

**Figure 2:**
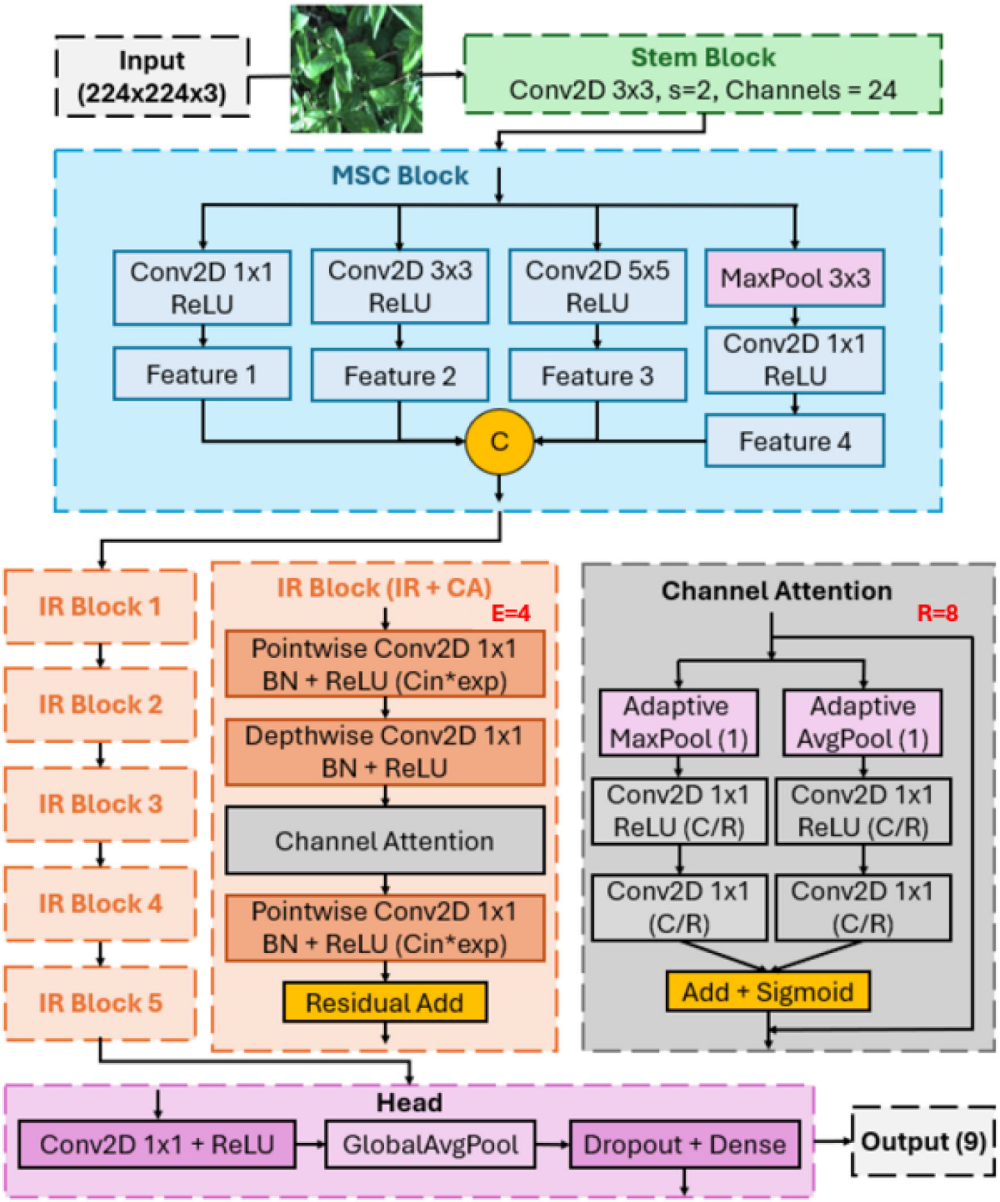
Architecture of TinyWeedNet showing the stem, multi-scale convolution (MSC) block with four parallel branches, inverted residual (IR) block with expansion ratio *E* = 4 and depthwise separable convolution, channel attention (CA) module with reduction ratio *R* = 8, and the classification head.

This section presents the architecture of TinyWeedNet, a lightweight CNN designed for real-time weed classification on low-power MCUs. As illustrated in **Figure 2**, TinyWeedNet adopts a hierarchical design comprising four core modules: a compact *Stem* for early feature extraction, a *Multi-Scale Convolution (MSC)* block for receptive-field diversity, a stack of *Inverted Residual (IR)* blocks enhanced with *Channel Attention (CA)*, and a minimalist classification head. A central design principle of TinyWeedNet is to maximize computational efficiency under the strict memory and latency constraints of MCU-based platforms. To this end, all network components are constructed exclusively from operators natively supported by the STM32Cube.AI^1^ framework including standard, pointwise, and depthwise convolutions, batch normalization, ReLU activation, pooling, and linear layers. This ensures full hardware compatibility, facilitates integer quantization, and enables memory-aware code generation for seamless embedded deployment.

Throughout this section, we denote the input image as $X\;\in \;{\mathbb{R}}^{3\times H\times W}$ with H=W=224, and a feature map at any stage as $F\;\in \;{\mathbb{R}}^{C\times H\times W}$. We use ${\text{Conv }}_{k\times k}^{s}$ and ${\text{DWConv }}_{k\times k}^{s}$ to represent standard and depthwise convolutions with kernel size k×k and stride s, respectively; BN (·) denotes batch normalization, σ(·) is the ReLU activation, and GAP (·) / GMP (·) represent global average and max pooling. These operations are consistently used across all modules described below. More details could be found in **Table 1**.

**Table 1:** TinyWeedNet layer schedule (input size 224×224).

Stage	Output size	Operator/Channels
Input	224×224	RGB
Stem	112×112	Conv 3×3, *C* = 24, *s* = 2
MSC	112×112	36 (concat of 1×1, 3×3, 5×5, pool+1×1)
IR#1	56×56	IR(*E* = 4, CA *R* = 8), 36→48, *s* = 2
IR#2	28×28	IR(*E* = 4, CA *R* = 8), 48→72, *s* = 2
IR#3	14×14	IR(*E* = 4, CA *R* = 8), 72→96, *s* = 2
IR#4	7×7	IR(*E* = 4, CA *R* = 8), 96→120, *s* = 2
IR#5	7×7	IR(*E* = 4, CA *R* = 8), 120→120, *s* = 1
Head	1×1	Conv 1×1: 120→240; GAP; Dropout; FC(9)

#### 2.2.1 Stem Block

The Stem Block (green section in **Figure 2**) serves as the entry point of TinyWeedNet, responsible for low-level feature extraction and spatial downsampling of the input image. It applies a single 3×3 convolution layer with stride s=2 and $C_{0}=24$ output channels, followed by batch normalization and a ReLU activation in Equation 1:

(1) $$F_{0}=\sigma \;\left(\text{BN}\left({\text{Conv}}_{3\times 3}^{s=2}\left(X\right)\right)\right),\;\;F_{0}\in {\mathbb{R}}^{24\times 112\times 112}.$$

This operation reduces the spatial resolution by a factor of two. The relatively narrow channel width (*C*_0_ = 24) ensures a lightweight feature representation that minimizes computation in subsequent layers while maintaining sufficient expressive capacity for early-stage encoding. Such an compact stem design is particularly beneficial for TinyML deployment, as it balances representational richness and on-chip efficiency, enabling fast inference on low-power MCUs.

#### 2.2.2 Multi-Scale Convolution block

The MSC block (**Figure 2** blue part) aims to enhance receptive-field diversity while maintaining computational efficiency. Natural scenes in agricultural environments often exhibit significant intra-class variations and complex backgrounds—e.g., overlapping leaves, varying weed sizes, and inconsistent lighting. To effectively handle these spatial complexities, the MSC employs four parallel convolutional branches with distinct receptive fields and a pooling pathway to capture features across multiple spatial scales.

Given the input feature map $F_{0}\in {\mathbb{R}}^{24\times 112\times 112}$ from the Stem block, the MSC block processes it through four concurrent branches shown in Equations 2–5:

(2) $$B_{1}=\sigma \left(\text{BN}\left({\text{Conv}}_{1\times 1}\left(F_{0}\right)\right)\right),$$

(3) $$B_{2}=\sigma \left(\text{BN}\left({\text{Conv}}_{3\times 3}^{s=1}\left(F_{0}\right)\right)\right),$$

(4) $$B_{3}=\sigma \left(\text{BN}\left({\text{Conv}}_{5\times 5}^{s=1}\left(F_{0}\right)\right)\right),$$

(5) $$B_{4}=\sigma \left(\text{BN}\left({\text{Conv}}_{1\times 1}\left({\text{MaxPool}}_{3\times 3}^{s=1}\left(F_{0}\right)\right)\right)\right).$$

Here, the first branch with 1 × 1 kernels performs local feature compaction and inter-channel mixing, the 3 × 3 and 5 × 5 branches extract mid- and large-scale spatial structures, and the fourth branch incorporates a 3×3 max-pooling operation to capture contextual cues such as background contrast before projection via a 1 × 1 convolution. All convolutional outputs are batch-normalized and activated using ReLU to ensure stable training and non-linear feature transformation.

Finally, the outputs of the four branches are concatenated along the channel dimension as follow in Equation 6:

(6) $$F_{\text{msc}}=\text{Concat}\left[B_{1},B_{2},B_{3},B_{4}\right]\in {\mathbb{R}}^{36\times 112\times 112}.$$

To maintain parameter uniformity across hardware deployments, the total output channels are fixed at 36, with each branch contributing roughly one quarter of the total (as determined using the _make_divisible function in the implementation). This design allows the MSC to capture both fine-grained edge patterns and coarse-scale weed morphology while preserving the same spatial resolution as the Stem output. These kernel scales were empirically selected to capture the diverse weed morphologies ranging from fine leaf veins to broad canopy textures. By aggregating multi-scale features early in the network, the subsequent layers can focus on high-level abstraction without sacrificing low-level texture fidelity—an essential property for visual tasks executed on resource-constrained MCUs.

#### 2.2.3 Inverted Residual block with channel attention

The Inverted Residual block constitutes the core feature transformation unit of TinyWeedNet, combining efficient depthwise separable convolutions with lightweight channel attention to achieve trade-off between high accuracy and efficiency (**Figure 2** orange part). Each IR block adopts an “expand–depthwise–project” pattern, which was first introduced in MobileNetV2 and later optimized for edge deployment Sandler et al. (2018). Given an input feature map $U\in {\mathbb{R}}^{C_{\text{in}}\times H\times W}$, the IR block expands the channel dimension by a factor of E=4, applies spatial filtering through a depthwise convolution, recalibrates the resulting features via a Channel Attention mechanism, and finally projects the output back to the target dimension $C_{\text{out}}$. The value of E is determined by the hyperparameter sensitivity analysis discussed later. Residual connections are included when the input and output tensors share identical shapes ( s=1 and $C_{\text{in}}=C_{\text{out}}$) to facilitate gradient propagation.

The complete transformation can be expressed as in Equations 7–10:

(7) $$Z_{e}=\sigma \;\left(\text{BN}\left({\text{Conv}}_{1\times 1}\left(U\right)\right)\right),$$

(8) $$Z_{d}=\sigma \;\left(\text{BN}\left({\text{DWConv}}_{3\times 3}^{s}\left(Z_{e}\right)\right)\right),$$

(9) $$Z_{a}={\text{CA}}_{R=8}\left(Z_{d}\right),$$

(10) $$V=\text{BN}\left({\text{Conv}}_{1\times 1}\left(Z_{a}\right)\right)),$$

where $Z_{e}\;\in \;{\mathbb{R}}^{\left(4C_{\text{in}}\right)\times H\times W}$, $Z_{d}\;\in \;{\mathbb{R}}^{\left(4C_{\text{in}}\right)\times \left(H/s\right)\times \left(W/s\right)}$, $V\;\in \;{\mathbb{R}}^{C_{\text{out}}\times \left(H/s\right)\times \left(W/s\right)}$.

When the stride s=1 and the channel dimensions remain unchanged, a residual connection is added shown in Equation 11:

(11) Y=U+V.

This shortcut enhances gradient flow and stabilizes the optimization process, allowing deeper feature extraction without accuracy degradation. By operating primarily in the channel domain, the IR block minimizes the computational cost associated with conventional full convolutions, reducing the number of multiply–accumulate operations while retaining representational richness.

To further improve feature discrimination, a lightweight CA (R = 8) mechanism is embedded after the depthwise stage in each IR block (**Figure 2** gray part). CA adaptively re-weights channel responses by modeling inter-channel dependencies using global pooling operations and a shared two-layer 1 × 1 convolutional bottleneck. The value of R is determined by the hyperparameter sensitivity analysis discussed later. For an input feature tensor $Z_{d}$, the attention response is compress as in Equations 12 and 13:

(12) $$q_{\text{avg}}=\text{GAP}\left(Z_{d}\right),\;q_{\text{max}}=\text{GMP}\left(Z_{d}\right),$$

(13) $$s=\sigma_{g}\;\left(W_{2}\;\sigma \left(W_{1}\;q_{\text{avg}}\right)+W_{2}\;\sigma \left(W_{1}\;q_{\text{max}}\right)\right),$$

where $W_{1}\in {\mathbb{R}}^{\frac{C}{R}\times C}$ and $W_{2}\in {\mathbb{R}}^{C\times \frac{C}{R}}$ are 1×1 convolutional layers functioning as an efficient two-layer perceptron, σ(·) denotes ReLU, and $\sigma_{g}\left(\cdot \right)$ is the sigmoid activation. The attended feature map is obtained as $Z_{a}=Z_{d}⊙s$, where ⊙ represents channel-wise multiplication. With a reduction ratio R=8, this mechanism imposes minimal overhead while enabling the network to emphasize weed-related features (e.g., leaf shape, vein contrast) and suppress background noise.

TinyWeedNet stacks five IR blocks sequentially, progressively reducing the spatial resolution while expanding channel depth. The first four IR blocks employ a stride of s=2 for spatial downsampling, and the final block keeps s=1 to preserve high-level spatial context.

This hierarchical design enables gradual abstraction from low-level spatial textures to high-level semantic representations, ensuring robust weed background separation under varying field conditions. The embedded CA modules at each stage further enhance intra-class compactness and inter-class separability, leading to improved classification robustness on resource-constrained devices.

#### 2.2.4 Final Projection and Classification Head

The Final Projection and Classification Head (**Figure 2** pink part) aggregates the high-level representations extracted by the preceding IR blocks and transforms them into class-level predictions. After the last IR stage, the feature tensor $F_{\text{IR}5}\in {\mathbb{R}}^{120\times 7\times 7}$ undergoes a 1×1 convolutional projection that expands the channel dimension to $C_{f}=240$ for enhanced representational richness shown in Equation 14:

(14) $$F_{f}=\sigma \;\left(\text{BN}\left({\text{Conv}}_{1\times 1}\left(F_{\text{IR}5}\right)\right)\right),\;F_{f}\in {\mathbb{R}}^{240\times 7\times 7}.$$

This 1 × 1 layer performs linear channel mixing and feature compression without altering spatial dimensions, allowing the network to consolidate multi-channel semantic descriptors from previous layers into a compact yet expressive feature space. Batch Normalization stabilizes the activation distribution, and the ReLU activation introduces non-linearity while preserving computational simplicity for MCU deployment.

Subsequently, global average pooling (GAP) aggregates the spatial information of $F_{f}$ into a 240-dimensional feature vector shown in Equation 15:

(15) $$p=\text{GAP}\left(F_{f}\right)\in {\mathbb{R}}^{240}.$$

This operation effectively converts each feature map into a single representative statistic, thus eliminating spatial redundancy and ensuring translation invariance—a desirable property for real-world weed imagery where plants appear at varying positions and scales.

To prevent overfitting and improve generalization, a dropout layer with a probability of p = 0.2 is applied before the final classifier. The resulting feature vector is then passed through a fully connected (FC) layer to produce the final prediction vector $\hat{y}$ over the nine target weed categories shown in Equation 16:

(16) $$\hat{y}=\text{FC}\left(\text{Dropout}\left(p\right)\right)\in {\mathbb{R}}^{9}.$$

This minimalistic classification head provides an trade-off between inference latency and predictive accuracy. By combining global pooling with a single dense layer, TinyWeedNet avoids the parameter overhead of multi-layer classifiers while maintaining discriminative capability. Such a streamlined design, together with integer-quantization compatibility, ensures that the network can be deployed on low-power STM32 MCUs for real-time weed classification tasks.

### 2.3 Experimental configuration and baseline models

All experiments were conducted on a high-performance computing platform running Ubuntu 22.04, featuring an Intel^®^ Core™ i9–12900 processor, 32GB DDR5 RAM, and an NVIDIA GeForce RTX 3090 GPU. The development environment consisted of VS Code IDE with Python 3.9.19 and PyTorch^2^ 2.8.0 framework. The training hyperparameters were as follows: a learning rate of 0.001, a batch size of 32, and 100 epochs with the SGD optimizer. The cross-entropy loss function was used, and an early stopping strategy was introduced to terminate training when the accuracy no longer improved, ensuring algorithm convergence.

To comprehensively evaluate the effectiveness of the proposed TinyWeedNet architecture, a comparison study was conducted against several state-of-the-art (SOTA) CNNs that are widely adopted for image classification tasks. The selected models represent diverse architectural paradigms, ranging from early compact designs to more advanced high-capacity networks. Specifically, the comparison includes SqueezeNet Iandola et al. (2016), GoogleNet Szegedy et al. (2016), VGG16 and VGG19 Simonyan and Zisserman (2014), ResNet18 and ResNet101 He et al. (2016), Inception v3 Szegedy et al. (2016), MobileNet V2 Sandler et al. (2018), Xception Chollet (2017), and EfficientNet B3 Tan et al. (2019). These models were selected to cover a broad spectrum of model sizes, depths, and computational complexities, providing a representative benchmark for assessing both accuracy and efficiency.

All networks were trained and tested under the same experimental protocol to ensure fairness of comparison. The input image size was fixed at 224 × 224 × 3, and identical data preprocessing was applied across all models. Each model was trained from beginning on the weed classification dataset for the same number of epochs, using the same learning-rate schedule and batch size. For lightweight architectures (e.g., MobileNetV2, SqueezeNet), default width multipliers were used, while for heavy architectures (e.g., ResNet101, EfficientNet-B3), depth and width configurations were kept standard without additional pruning.

The performance metrics considered include classification accuracy, F1-score, and parameter count, which jointly reflect prediction performance, and model complexity. All experiments were repeated ten times, and the mean values with their standard deviations (mean ± SD) were reported.

### 2.4 MCU deployment framework

**Figure 3:**
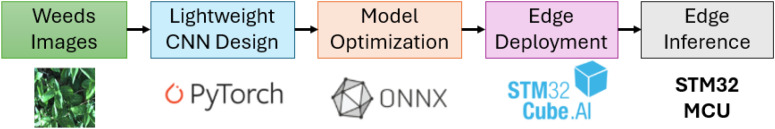
Tiny machine learning workflow.

To achieve on-device weed classification, the optimized TinyWeedNet model is deployed on an STM32 MCU following the standardized Tiny Machine Learning workflow, as illustrated in **Figure 3**. The deployment process involves four main stages: (1) model design and training using PyTorch; (2) format conversion and optimization through the Open Neural Network Exchange^3^ (ONNX) intermediate representation; (3) conversion to MCU-executable code via STM32Cube.AI; and (4) model integration and inference execution on embedded hardware. After model training, the PyTorch checkpoint is exported to ONNX as a framework-independent representation, which is then imported into STM32Cube.AI for automatic graph optimization and layer fusion. The tool subsequently generates ANSI C source code with fixed-point parameters, including initialization functions and inference routines that can be compiled within STM32CubeIDE. This workflow ensures seamless transition from high-level model development to resource-constrained deployment, enabling efficient, fully integer-based inference execution with memory-aware scheduling on embedded targets.

The model deployment and evaluation were conducted on an STM32H7B3I-EVAL development board. The main hardware specifications are summarized in **Table 2**. All experiments were performed at 3.3 V low-dropout (LDO) regulation mode, which allows power consumption measurement through the onboard current-sensing interface. Internal SRAM and flash memory were prioritized for model deployment and execution, while external memory was utilized only when internal storage capacity was insufficient.

**Table 2:** Technical specifications of STM32H7B3I-EVAL.

Parameter	STM32H7B3I-EVAL development board
MCU	STM32H7B3LIH6Q
CPU Core	ARM Cortex M7 with FPU
CPU Frequency	280MHz
RAM	1.4 MB internal + 16 MB external SRAM
Flash	2 MB internal + 64 MB external flash
Voltage	3.3V
Operating Current	132.5mA (Low-Dropout Regulator mode)

Model integration, code compilation, and firmware flashing were performed in STM32-CubeIDE using C++ as the implementation language. The generated inference code was linked with the STM32 HAL library and executed on bare-metal firmware without an operating system. Ten images were stored in array format on the MCU as model inputs for on-device performance testing. Model accuracy was tested on the PC using the ONNX framework.

This deployment framework provides a reusable toolchain for converting TinyWeedNet from a high-level deep learning model to a fully functional TinyML implementation on an MCU. It also establishes a unified environment for subsequent measurements of latency, memory usage, and energy consumption under real-world embedded conditions.

### 2.5 Hyperparameter exploration

To systematically explore the trade-off between model accuracy and deployment efficiency, a hyperparameter analysis was conducted focusing on three key architectural parameters of TinyWeedNet: the Expand Ratio, the Reduction Ratio, and the Stem Channels. The selection of these three hyperparameters was guided by both architectural significance and deployment practicality. These hyperparameters control the expansion width in IR blocks, the compression factor in CA modules, and the number of feature maps in the initial stem convolution layer, respectively.

For each hyperparameter, a discrete search space was defined based on empirical design constraints and MCU memory limits:

Expand Ratio E∈{3, 4, 6}Reduction Ratio R∈{4, 8, 16}Stem Channels S∈{8, 16, 24}A full factorial combination experiment was performed, resulting in a total of 3×3×3 = 27 model variants. All configurations were trained using the same optimization setup and evaluated on both accuracy-oriented and deployment-oriented metrics, including classification accuracy, F1-score, number of parameters, model size, and inference latency. To ensure fairness, each configuration was trained from scratch under identical random seeds and training epochs, and early stopping was applied based on the model’s accuracy on the test set.

The *Expand Ratio (E)* directly controls the intermediate channel width within the inverted residual (IR) blocks, thereby influencing the network’s representational capacity and computational cost. Lower values (e.g., *E* = 3) favor compactness and lower latency, whereas higher values (e.g., *E* = 6) enhance feature richness at the expense of larger parameter counts. The *Reduction Ratio (R)* in the CA module determines the degree of channel compression and thus governs the balance between feature selectivity and overhead in the attention subnetwork. A moderate reduction ratio (*R* = 8) was hypothesized to offer the best trade-off between discriminative capability and MCU efficiency. Finally, the *Stem Channels (S)* parameter controls the number of filters in the initial convolution layer, affecting both early feature diversity and the propagation of representational capacity throughout the network.

The discrete ranges of E, R, and S were selected based on prior empirical findings from lightweight CNNs such as MobileNet and EfficientNet, as well as hardware profiling constraints observed on the STM32H7 platform. Values beyond these ranges (e.g., E>6 or S>24) led to significant memory overflow or increased inference latency without measurable accuracy gain in preliminary tests. A full-factorial exploration was therefore adopted to comprehensively assess the interdependence among these factors and to ensure that the final configuration, later identified as Combo15, represents a globally optimal design rather than a locally tuned solution.

The quantitative outcomes and deployment performance associated with all hyperparameter configurations are analyzed in Section 3, where the trade-offs between accuracy, latency, and energy consumption are discussed in detail.

### 2.6 Ablation study design

To evaluate the contribution of each key component within the proposed TinyWeedNet architecture, a series of ablation experiments were conducted. Each experiment selectively disables or replaces a specific module while keeping all other training and evaluation settings identical to the baseline configuration. This allows for isolating the impact of individual design choices on both model accuracy and deployment performance.

Four ablation variants were designed by removing one specific module from the original model as follows:

- The Multi-Scale Convolution module is replaced with a single 3 × 3 convolution branch, removing the parallel receptive-field aggregation mechanism.
- All Channel Attention modules are removed from the IR blocks, disabling adaptive feature recalibration.
- Depthwise Convolution convolutions are replaced with standard 3 × 3 convolutions, resulting in higher computational cost but unchanged spatial topology.
- The Final Conv1×1 projection layer before global average pooling is omitted, and the feature maps are directly fed into the GAP–FC head.

Each simplified model was retrained from scratch using the same optimization settings, data preprocessing pipeline, and hyperparameter configuration as the baseline TinyWeedNet model. The performance was then evaluated using identical metrics, including classification accuracy, F1-score, number of parameters, model size, inference latency, and energy per inference.

### 2.7 Robustness analysis design under controlled domain shifts

To evaluate the robustness of the proposed model under realistic environmental variations without introducing additional datasets, a controlled robustness analysis is conducted on the DeepWeeds test set. Rather than assessing cross-dataset generalization, which would require out-of-distribution data, this analysis focuses on quantifying the model’s sensitivity to appearance changes commonly encountered in field deployment scenarios, including illumination variations, weather-induced image degradation, and soil or background color shifts.

Let $I\in {\mathbb{R}}^{H\times W\times 3}$ denote a clean RGB test image, and let f(·) represent the trained classification model. For each robustness experiment, a perturbed image $I^{\prime }$ is generated by applying a transformation T(·;θ), while keeping the ground-truth label unchanged.

#### 2.7.1 Illumination variations

Illumination changes are simulated using four photometric transformations: brightness adjustment, contrast scaling, gamma correction, and white-balance shift.

Brightness adjustment is defined as in Equation 17.

(17) $$I^{\prime }=\text{clip}\left(I+\beta \right),$$

where β∈{0.10, 0.20, 0.30} controls the intensity of brightness variation after image normalization to [0,1]. These values are selected to represent mild to strong exposure changes commonly observed in outdoor agricultural environments.

Contrast scaling is formulated as in Equation 18.

(18) $$I^{\prime }=\text{clip}\left(\alpha \left(I-\mu \right)+\mu \right),$$

where μ denotes the mean pixel intensity and α∈{0.8, 0.6, 0.4} represents progressively decreasing contrast levels. This range captures gradual contrast degradation caused by overcast weather or sensor limitations while avoiding unrealistic visual artifacts.

Gamma correction is applied as in Equation 19.

(19) $$I^{\prime }=I^{\gamma },$$

with γ∈{0.8, 1.2, 1.6}. These values are chosen to approximate non-linear illumination effects under shadowed and high-glare conditions frequently encountered in field imaging.

White-balance shift is modeled through channel-wise scaling as in Equation 20:

(20) $${I^{\prime }}_{c}=s_{c}\cdot I_{c},\;c\in \left\{R,\;G,B\right\},$$

where $\left(s_{R},s_{G},s_{B}\right)\in \left\{\left(1.1,1.0,0.9\right),\left(1.2,1.0,0.8\right),\left(1.3,1.0,0.7\right)\right\}$. These settings simulate color temperature variations caused by different sunlight spectra throughout the day.

#### 2.7.2 Weather-induced degradation

Weather-related image degradation is approximated using Gaussian blur, which models reduced image sharpness due to motion, defocus, light rain, or atmospheric haze.

Gaussian blur is defined as in Equation 21.

(21) $$I^{\prime }=I*G_{\sigma },$$

where $G_{\sigma }$ denotes a Gaussian kernel. Kernel sizes of {3, 5, 7} are employed to represent increasing levels of degradation severity. These values are selected to reflect realistic blur conditions encountered by mobile agricultural platforms, while preserving the semantic structure of the target objects.

#### 2.7.3 Soil and background color variation

To approximate variations in soil appearance and background color distributions without introducing new textures or datasets, a hue shift is applied in the HSV color space as in Equation 22:

(22) $$H^{\prime }=H+\text{Δ}h,$$

where $\text{Δ}h\in \left\{\pm 5^{\circ },\pm 10^{\circ },\pm 15^{\circ }\right\}$. This perturbation serves as a lightweight proxy for changes in soil color, vegetation background, and regional color distributions, enabling an initial robustness assessment under controlled background-related domain shifts.

## 3 Results and discussion

### 3.1 CNN benchmark comparison

**Figure 4:**
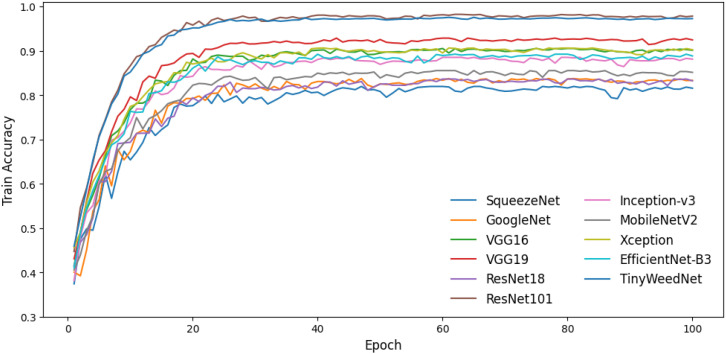
Training accuracy curves of different CNN models over 100 epochs.

As shown in **Table 3**; **Figure 4**, TinyWeedNet achieves competitive classification accuracy (97.26%) and F1-score (96.64%), comparable to deeper architectures such as ResNet101 while using only 0.48M parameters. This represents a parameter reduction of approximately two orders of magnitude compared to VGG- and ResNet-family models, highlighting the efficiency of the proposed design. Although ResNet101 achieved a slightly higher accuracy (97.82%), TinyWeedNet achieved 200× higher accuracy-per-parameter efficiency (0.97/0.48M vs. 0.97/42.5M). Unlike large models that rely on extensive channel widths and deep hierarchies, TinyWeedNet achieves strong discriminative capability through its compact combination of multi-scale convolutions, depthwise separable operations, and lightweight channel attention mechanisms.

**Table 3:** Comparison of classification performance and model complexity.

Model	Accuracy (%)	F1-score (%)	Params. (M)
SqueezeNet	81.55 ± 0.87	81.98 ± 0.76	0.7
GoogleNet	83.33 ± 0.45	83.64 ± 0.41	12.0
VGG16	90.12 ± 0.25	90.33 ± 0.22	134.3
VGG19	92.45 ± 0.49	94.48 ± 0.41	139.6
ResNet18	83.22 ± 0.91	83.55 ± 0.78	25.6
ResNet101	97.82 ± 0.66	97.76 ± 0.43	42.5
Inception-v3	88.11 ± 0.48	88.33 ± 0.58	21.8
MobileNetV2	85.13 ± 0.34	84.46 ± 0.44	2.2
Xception	90.23 ± 0.76	89.56 ± 0.41	25.6
EfficientNet-B3	88.83 ± 0.98	87.85 ± 0.77	11.2
**TinyWeedNet**	97.26 ± 0.72	96.64 ± 0.76	0.4758

RED and BLUE indicate the first and second highest ranking results, respectively. BOLD indicates the proposed model.

In contrast, other lightweight networks (e.g., SqueezeNet and MobileNetV2) demonstrate accuracy values below 86%, indicating limited feature representation power for complex natural scenes. TinyWeedNet therefore provides a more balanced trade-off between accuracy and compactness, making it especially suitable for deployment in resource-constrained edge devices.

Overall, the benchmark results demonstrate that TinyWeedNet achieves near state-of-the-art accuracy with the lowest model complexity among all tested CNNs. This performance–efficiency synergy confirms its suitability for embedded weed recognition tasks and supports its deployment on MCU-based TinyML platforms.

### 3.2 Hyperparameter sensitivity analysis

**Table 4** summarizes the joint influence of three key architectural hyperparameters, the Expand Ratio, Reduction Ratio, and Stem Channels, on both classification performance and MCU deployment efficiency. A total of 27 network configurations were systematically trained and evaluated to investigate how these factors affect representational capacity, computational complexity, and resource utilization. These experiments were designed to validate the effectiveness of the proposed TinyWeedNet configuration (E = 4, R = 8, and S = 24), which was originally derived from theoretical design considerations and preliminary observations on model scaling.

**Table 4:** Hyperparameter sensitivity analysis and On-MCU performance across combined configurations: Expand Ratio (E), Reduction Ratio (R), and Stem Channels (S).

ID	Experiment	Params.	Model size (MB)	Accuracy (%)	F1-score (%)	Inf. time (ms)	MACC	Flash (int.)	RAM (int. + ext.)	Energy/Inf. (mJ)
1	E3_R4_S8	51.5K	0.196	88.55 ± 0.65	83.92 ± 0.72	12.67 ± 0.22	3.98 × 10^7^	236.4 KiB	24.1 KiB + 2.34 MiB	5.54
2	E3_R4_S16	187.5K	0.715	95.72 ± 0.83	94.32 ± 0.55	31.65 ± 0.40	1.10 × 10^8^	762.9 KiB	24.3 KiB + 3.51 MiB	13.82
3	E3_R4_S24	416.3K	1.588	96.55 ± 0.91	95.47 ± 0.73	73.45 ± 1.24	2.64 × 10^8^	1.61 MiB	24.3 KiB + 5.85 MiB	32.08
4	E3_R8_S8	43.2K	0.165	89.32 ± 0.58	85.42 ± 0.46	12.79 ± 0.36	3.97 × 10^7^	203.8 KiB	24.2 KiB + 2.34 MiB	5.59
5	E3_R8_S16	155.1K	0.592	95.49 ± 0.68	94.03 ± 0.66	31.48 ± 0.28	1.10 × 10^8^	636.3 KiB	24.4 KiB + 3.51 MiB	13.76
6	E3_R8_S24	342.8K	1.308	96.69 ± 0.84	95.40 ± 0.59	74.05 ± 0.29	2.64 × 10^8^	1.33 MiB	24.4 KiB + 5.85 MiB	32.34
7	E3_R16_S8	38.8K	0.148	87.18 ± 0.44	81.78 ± 0.55	12.80 ± 0.13	3.97 × 10^7^	186.6 KiB	24.2 KiB + 2.34 MiB	5.60
8	E3_R16_S16	138.8K	0.530	94.32 ± 0.73	92.26 ± 0.61	32.09 ± 0.39	1.10 × 10^8^	572.8 KiB	24.4 KiB + 3.51 MiB	14.03
9	E3_R16_S24	305.3K	1.165	96.40 ± 0.66	95.44 ± 0.58	74.94 ± 0.79	2.64 × 10^8^	1.19 MiB	24.4 KiB + 5.85 MiB	32.75
10	E4_R4_S8	74.2K	0.283	91.24 ± 0.56	88.11 ± 0.69	15.39 ± 0.16	4.71 × 10^7^	322.8 KiB	23.9 KiB + 3.12 MiB	6.72
11	E4_R4_S16	272.4K	1.039	95.83 ± 0.91	94.36 ± 0.64	38.15 ± 0.92	1.31 × 10^8^	1.07 MiB	24.1 KiB + 4.69 MiB	16.67
12	E4_R4_S24	606.6K	2.314	96.95 ± 0.77	95.98 ± 0.81	87.80 ± 0.85	3.13 × 10^8^	2.33 MiB (ext.)	24.1 KiB + 7.81 MiB	38.41
13	E4_R8_S8	59.3K	0.226	91.49 ± 0.48	88.29 ± 0.67	15.53 ± 0.07	4.71 × 10^7^	265.2 KiB	24.1 KiB + 3.12 MiB	6.79
14	E4_R8_S16	214.8K	0.819	95.35 ± 0.79	93.67 ± 0.68	38.02 ± 0.64	1.31 × 10^8^	866.6 KiB	24.3 KiB + 4.69 MiB	16.61
15	E4_R8_S24	475.8K	1.815	97.26 ± 0.72	96.64 ± 0.76	89.40 ± 1.00	3.12 × 10^8^	1.83 MiB	24.4 KiB + 7.81 MiB	39.08
16	E4_R16_S8	51.9K	0.198	88.98 ± 0.61	84.95 ± 0.53	15.04 ± 0.64	4.71 × 10^7^	236.3 KiB	24.2 KiB + 3.12 MiB	6.58
17	E4_R16_S16	186.0K	0.710	95.43 ± 0.69	94.11 ± 0.71	38.49 ± 0.26	1.31 × 10^8^	754.3 KiB	24.4 KiB + 4.69 MiB	16.81
18	E4_R16_S24	410.4K	1.565	96.23 ± 0.81	94.90 ± 0.65	86.43 ± 0.68	3.13 × 10^8^	1.59 MiB	24.4 KiB + 7.81 MiB	37.79
19	E6_R4_S8	130.6K	0.498	94.58 ± 0.55	92.79 ± 0.78	21.31 ± 0.28	6.19 × 10^7^	541.0 KiB	24.1 KiB + 4.69 MiB	9.32
20	E6_R4_S16	485.5K	1.852	96.80 ± 0.82	95.85 ± 0.91	56.68 ± 0.42	1.71 × 10^8^	1.87 MiB	24.4 KiB + 7.03 MiB	24.77
21	E6_R4_S24	1085.2K	4.140	97.29 ± 0.69	96.42 ± 0.89	118.34 ± 1.37	4.10 × 10^8^	4.15 MiB (ext.)	24.4 KiB + 11.72 MiB	51.72
22	E6_R8_S8	97.2K	0.371	93.23 ± 0.59	90.73 ± 0.67	19.83 ± 0.24	6.18 × 10^7^	410.2 KiB	24.1 KiB + 4.69 MiB	8.66
23	E6_R8_S16	355.9K	1.358	96.32 ± 0.74	95.21 ± 0.65	49.91 ± 0.62	1.71 × 10^8^	1.38 MiB	24.3 KiB + 7.03 MiB	21.81
24	E6_R8_S24	790.9K	3.017	97.00 ± 0.88	96.18 ± 0.77	115.56 ± 1.67	4.10 × 10^8^	3.03 MiB (ext.)	24.4 KiB + 11.72 MiB	50.51
25	E6_R16_S8	80.5K	0.307	93.75 ± 0.47	91.42 ± 0.68	21.39 ± 0.24	6.18 × 10^7^	344.9 KiB	24.2 KiB + 4.69 MiB	9.35
26	E6_R16_S16	291.1K	1.110	95.83 ± 0.79	94.58 ± 0.66	51.80 ± 0.90	1.71 × 10^8^	1.13 MiB	24.4 KiB + 7.03 MiB	22.64
27	E6_R16_S24	643.7K	2.456	96.40 ± 0.91	95.26 ± 0.81	118.93 ± 1.76	4.10 × 10^8^	2.47 MiB (ext.)	24.4 KiB + 11.72 MiB	51.98

RED and BLUE indicate the first and second highest ranking results, respectively.

#### 3.2.1 Effect of expand ratio

The expansion ratio *E* determines the internal channel width within each IR block, thus shaping the model’s feature capacity and non-linearity. When *E* increases from 3 to 6, both accuracy and F1-score improve steadily because wider intermediate layers capture more diverse spatial patterns. However, this benefit comes with a steep rise in computational cost and memory usage. For example, the configuration with *E* = 6 attains the best accuracy (97.29%) but incurs a ninefold increase in inference latency (118.34 ms versus 12.67 ms) and a 26-fold rise in flash memory compared with *E* = 3. Beyond a certain point, further expansion yields little accuracy gain but severely limits deployability on MCUs. In practice, a moderate setting of *E* = 4 offers the best balance—achieving 96–97% accuracy while keeping inference time below 90 ms and flash usage under 2 MB. This trend echoes prior findings on lightweight vision networks Sandler et al. (2018), where moderate expansion preserves accuracy without sacrificing efficiency.

#### 3.2.2 Effect of reduction ratio

The reduction ratio *R* in the channel attention (CA) module governs the degree of channel compression within the squeeze–excitation branch. A small *R* (e.g., 4) retains more intermediate channels and strengthens attention responses to subtle inter-class variations such as leaf texture or vein details. Yet, this comes at the expense of a larger parameter count and memory demand—for instance, increasing from 38.8 K parameters at *R* = 16 to 51.5 K at *R* = 4. In contrast, a large *R* (e.g., 16) overly compresses the feature descriptors, leading to minor but consistent accuracy drops (up to 2–3%). Across all configurations, *R* = 8 provides the most balanced outcome: F1-scores remain above 94% while parameters are reduced by roughly one quarter compared to *R* = 4. Configurations with *R* = 8 also show fast inference (12–115 ms) and stable energy use, confirming that moderate compression achieves the best trade-off between attention expressiveness and MCU-level efficiency. This observation further validates the lightweight attention strategy adopted in TinyWeedNet.

#### 3.2.3 Effect of stem channels

The number of stem channels *S* controls the capacity of the initial convolution to extract low-level edges and texture features. Increasing *S* from 8 to 24 leads to clear improvements in accuracy and F1-score (typically 1–3%), as richer early representations enhance gradient flow and feature discrimination. However, these benefits come with quadratic growth in both parameters and flash memory, from 0.196 MiB to 1.588 MiB, and require more data to be stored externally (up to 7.81 MiB), which adds energy overhead. Empirically, *S* = 24 represents a practical upper bound, offering strong feature extraction while staying within the 2 MB on-chip flash limit. Configurations beyond *E* = 6 or *S* = 24 exceeded STM32H7 storage capacity, emphasizing that architecture design in TinyML must be co-optimized with hardware constraints.

#### 3.2.4 Overall validation of the proposed configuration

When jointly considering accuracy, latency, and on-chip memory limits, the configuration (E4 R8 S24) defining the proposed TinyWeedNet architecture demonstrates the most balanced performance among all 27 tested combinations. It achieves 97.26% accuracy and 96.64% F1-score while maintaining a moderate model size (1.815 MB) and an inference latency (89.40 ms) for agricultural robotic vision systems. Unlike typical brute-force neural architecture searches, this configuration was *a priori* derived from design principles emphasizing multi-scale diversity, moderate expansion, and lightweight attention. The subsequent sensitivity experiments validate that this combination yields the best accuracy–efficiency–energy synergy, with per-inference energy of 39.08 mJ - 8× lower than the heaviest configuration (ID 21). Furthermore, the relatively small standard deviations across all trials (≤1%) indicate statistical robustness, confirming that TinyWeedNet’s performance remains stable under random initialization and environmental noise.

Overall, the hyperparameter sensitivity analysis not only verifies TinyWeedNet’s architectural choices but also provides generalizable insights for TinyML model design. In particular, (1) moderate expansion (E = 4-5) prevents accuracy saturation while ensuring low latency, (2) mid-level attention reduction (R = 8) balances precision and overhead, and (3) a strong but bounded stem (S = 24) offers maximal texture sensitivity without memory overflow. These findings collectively demonstrate that the TinyWeedNet configuration is hardware-aware, energy-efficient, and empirically validated for MCU deployment in weed classification scenarios.

### 3.3 Ablation study results

**Table 5**; **Figure 5** presents the ablation study results conducted to quantify the contribution of each architectural component in TinyWeedNet, including the multi-scale convolution, CA, depthwise separable convolution, and final 1 × 1 projection layer. All variants were retrained under identical settings to isolate the effect of individual modules on both predictive accuracy and MCU deployment metrics.

**Table 5:** Ablation study of the proposed TinyWeedNet on model architecture and MCU deployment performance.

Experiment	Params.	Model size	Accuracy (%)	F1-score (%)	Inf. time (ms)	MACC	Flash (int.)	RAM (int. + ext.)	Energy/Inf. (mJ)
Baseline	475K	1.81 MB	97.26 ± 0.72	96.64 ± 0.76	89.345 ± 1.021	3.12×10^8^	1.83 MiB	24.4 KiB + 7.81 MiB	39.08
w/o MS Conv	475K	1.81 MB	94.29 ± 0.67	91.96 ± 0.55	89.821 ± 0.606	3.12×10^8^	1.83 MiB	18.56 KiB + 7.81 MiB	39.23
w/o Attention	820K	3.13 MB	93.14 ± 0.78	90.75 ± 0.69	86.893 ± 0.661	3.10×10^8^	3.15 MiB (ext.)	10.88 KiB + 7.81 MiB	38.00
w/o DepthwiseConv	5.10M	19.72 MB	96.68 ± 0.92	95.58 ± 0.86	593.423 ± 4.960	1.60×10^9^	19.74 MiB (ext.)	18.95 KiB + 7.81 MiB	259.16
w/o Final Conv1×1	445K	1.69 MB	94.69 ± 0.71	92.78 ± 0.59	88.577 ± 0.830	3.10×10^8^	1.71 MiB	17.94 KiB + 7.81 MiB	38.69

RED and BLUE indicate the first and second highest ranking results, respectively.

**Figure 5:**
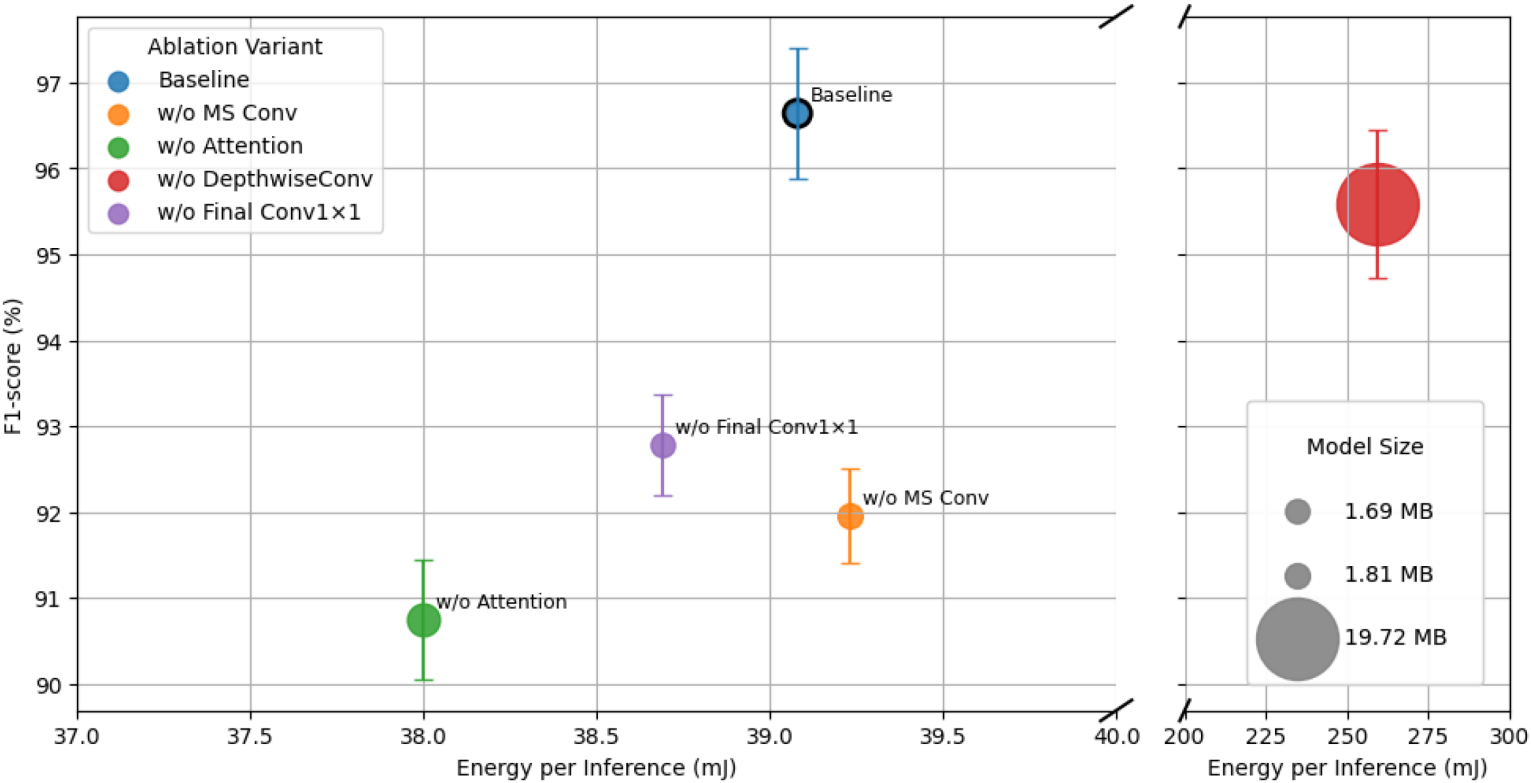
Trade-off between F1-score and energy consumption per inference for different TinyWeedNet ablation variants on the target MCU. Bubble size represents the model size. The x-axis is broken to highlight the low-energy region (30–40 mJ), while the high-energy outlier (w/o DepthwiseConv) is shown separately.

#### 3.3.1 Impact of multi-scale convolution

Removing the multi-scale convolution branch (“w/o MS Conv”) leads to a drop in accuracy (from 97.26% to 94.29%) and F1-score (from 96.64% to 91.96%), confirming that the multi-scale design (integrating kernels of 1 × 1, 3 × 3, and 5 × 5) is essential for capturing spatial features at multiple receptive-field resolutions. Interestingly, both inference time and energy consumption remain almost unchanged (Δ0.48 ms, Δ0.15 mJ), implying that the MS block introduces negligible computational cost relative to its gain in representational richness. In terms of parameter efficiency, the baseline achieves 204.8%/M accuracy-per-parameter (APP), while “w/o MS Conv” drops to 198.5%/M, highlighting that MS not only improves raw accuracy but also increases accuracy density per parameter. The energy-per-MACC also remains stable (≈1.25×10^−10^ J/MACC), demonstrating that the gain is architectural rather than computational.

#### 3.3.2 Impact of channel attention

Disabling the channel attention mechanism (“w/o Attention”) decreases F1-score to 90.75%, illustrating a loss of discriminative ability in feature reweighting. Although inference becomes slightly faster (86.9 ms) with lower energy consumption (38.0 mJ), this accuracy–efficiency trade-off is unfavorable for real deployments. Moreover, the variant unexpectedly increases model parameters (820K vs. 475K), confirming that the lightweight CA module not only strengthens feature expressiveness but also imposes a regularizing effect, allowing the backbone to remain compact without losing performance. From an energy–delay perspective, the Energy–Delay Product (EDP) improves only marginally (3.30×10^3^ vs. 3.49×10^3^ mJ·ms), while accuracy drops significantly, making the baseline configuration more Pareto-optimal. Overall, the CA module with reduction ratio *R* = 8 offers the best synergy between attention strength and hardware efficiency, ensuring both high accuracy and stable runtime.

#### 3.3.3 Impact of depthwise separable convolution

Replacing all depthwise separable convolutions with standard 3×3 convolutions (“w/o DepthwiseConv”) results in a catastrophic increase in parameter count from 0.48 M to 5.10 M, inflating model size over tenfold (1.8 MB → 19.7 MB) and MACC operations by 5.13×. While the accuracy remains high (96.68%), the inference latency rises from 89.3 ms to 593.4 ms, and energy consumption surges from 39.1 mJ to 259.2 mJ. This translates to a 44× increase in EDP (1.54 × 105 mJ·ms), making this variant less attractive for MCU applications. The per-MACC energy also deteriorates (0.162 nJ/MACC vs. 0.125 nJ/MACC), implying that standard convolutions magnify memory traffic and data-movement overhead. This confirms that depthwise separable convolutions are the dominant contributor to TinyWeedNet’s computational efficiency, maintaining high accuracy while minimizing latency and energy cost.

#### 3.3.4 Impact of final 1×1 Projection Layer

Removing the final 1 × 1 projection layer before global average pooling (“w/o Final Conv1×1”) leads to an accuracy reduction from 97.26% to 94.69% with a slight decrease in model size (1.81 MB → 1.69 MB). This projection consolidates high-level semantic features and improves inter-class separability prior to classification. Its computational overhead is minimal (0.3ms difference) and energy consumption remains virtually unchanged (38.7 mJ), demonstrating that its inclusion provides substantial accuracy gains with negligible runtime penalty. Although its accuracy-per-parameter (212.8%/M) seems higher numerically due to the reduced parameter count, the absolute performance loss of over 2.5% indicates that this layer is essential for final-stage feature fusion and stable convergence.

#### 3.3.5 Cross-module interactions and deployment implications

The ablation experiments also reveal non-linear interactions among components:

- Multi-Scale Convolutions↔Channel Attention synergy: The multi-scale features enhance the effectiveness of channel attention by providing diverse frequency and scale cues. Removing either module causes a super-linear drop (4–6% in F1-score) despite unchanged latency, underscoring their coupled role in maintaining feature robustness.
- Depthwise Convolutions↔Memory hierarchy: Depthwise convolutions help keep intermediate feature maps within on-chip SRAM, avoiding off-chip DRAM transfers. Once replaced by standard convolutions, the model exceeds internal flash limits (19.7 MB), forcing partial external memory access and introducing severe DMA stalls that amplify energy cost beyond what MACC scaling alone predicts.
- Projection↔Classifier alignment: The final 1 × 1 layer acts as a semantic bottleneck aligning channel responses for the classifier head. Its absence reduces F1-score by nearly 4% with minimal runtime impact, confirming its role as a structural regularizer rather than a compute burden.

These interactions highlight that removing modules based solely on FLOP reduction can be misleading in TinyML contexts; instead, optimizing for on-chip memory residency, energy-per-MACC stability, and accuracy-per-parameter density yields better deployment efficiency.

### 3.4 Robustness analysis results

**Table 6** summarizes the robustness performance of the proposed model on the DeepWeeds test set under controlled domain shifts. Overall, the model exhibits stable behavior under moderate perturbations, with a gradual and bounded degradation in F1-score as the severity level increases.

**Table 6:** TinyWeedNet robustness evaluation on the DeepWeeds test set under controlled domain shifts.

Corruption Type	Severity	F1-score (%)	ΔF1-score (%)
Clean	–	96.1	0.0
Illumination (Brightness)	1	95.7	-0.4
	2	95.0	-1.1
	3	94.0	-2.1
Illumination (Contrast)	1	95.4	-0.7
	2	94.5	-1.6
	3	93.1	-3.0
Illumination (Gamma)	1	95.5	-0.6
	2	94.6	-1.5
	3	93.4	-2.7
Illumination (White balance)	1	95.3	-0.8
	2	94.2	-1.9
	3	92.8	-3.3
Weather (Gaussian blur)	1	94.8	-1.3
	2	93.5	-2.6
	3	91.5	-4.6
Soil/background proxy (Hue shift)	1	95.6	-0.5
	2	94.8	-1.3
	3	93.8	-2.3

Severity levels 1, 2, and 3 correspond to mild, moderate, and severe perturbations, respectively, with increasing transformation strength for illumination variations, Gaussian blur, and background color shifts.

For illumination-related variations, including brightness, contrast, gamma correction, and white-balance shifts, the performance degradation remains limited. Across all illumination perturbations, the maximum F1-score drop at the most severe level (Severity 3) does not exceed 3.3 percentage points. In particular, brightness and gamma adjustments result in relatively small performance changes, indicating that the model is robust to common exposure fluctuations and non-linear illumination effects encountered in outdoor agricultural environments. White-balance shifts introduce slightly larger degradation, suggesting increased sensitivity to extreme color temperature variations.

Weather-induced degradation modeled by Gaussian blur leads to the largest performance decrease among all tested perturbations. At Severity 3, the F1-score drop reaches 4.6 percentage points, reflecting the challenge posed by reduced image sharpness due to motion, defocus, or adverse weather conditions. Nevertheless, the model maintains a high absolute F1-score of 91.5% even under severe blur, indicating a reasonable level of robustness in visually degraded scenarios.

Background-related variations approximated by hue shifts result in relatively minor performance degradation. Even at the highest severity level, the F1-score drop is limited to 2.3 percentage points, suggesting that the model is not overly sensitive to moderate changes in soil appearance or background color distributions. This behavior is desirable for deployment across different agricultural fields with varying soil and vegetation characteristics.

In summary, the robustness analysis demonstrates that the proposed model maintains consistent classification performance under a range of controlled appearance variations, with the most significant sensitivity observed under severe blur conditions. These results indicate that the model is well-suited for real-world agricultural deployment where illumination and background variations are common, while also highlighting weather-induced image degradation as a key factor for future improvement.

## 4 Conclusions and future work

In this study, we developed TinyWeedNet, a hardware-efficient lightweight CNN designed for real-time on-device weed identification in precision agriculture. Through a TinyML deployment workflow, the model was successfully implemented on an STM32H7 microcontroller, achieving 97.26% accuracy on the DeepWeeds dataset with sub-90 ms inference latency and an average energy cost of approximately 39 mJ per prediction. These results confirm that lightweight neural architectures, when carefully co-designed with hardware constraints, can support robust and responsive weed identification on low-power embedded platforms.

Comprehensive benchmark comparisons, hyperparameter sensitivity analyses, and ablation experiments further revealed how architectural factors, including expansion ratio, channel attention reduction, and stem width, shape performance under resource limitations. The configuration using a moderate expansion ratio (E = 4), compact attention reduction (R = 8), and a 24-channel stem achieved the best balance between accuracy, computational efficiency, and energy consumption. Ablation findings emphasized the importance of multi-scale feature extraction and lightweight attention mechanisms for maintaining high recognition performance, while highlighting the significant cost of removing depthwise separable convolutions. Together, these results underscore the value of hardware-aware architectural design in developing practical TinyML models for agricultural applications.

Beyond model development, this work contributes a reproducible evaluation methodology grounded in physically measured metrics (accuracy, latency, memory usage, and energy per inference). Such measurement-driven characterization provides an actionable energy–accuracy–latency map that can guide the design and optimization of embedded vision systems for autonomous field robots, UAVs, and distributed sensing nodes in smart farming.

The compact footprint and low power demand of TinyWeedNet make it particularly suitable for long-term, battery-powered or energy-harvesting agricultural deployments, where continuous in-field operation is required without reliance on cloud connectivity. Future research will extend the present work along several complementary directions to further enhance the practical applicability of TinyWeedNet in real-world agricultural deployments. First, a comprehensive cross-platform evaluation will be conducted across heterogeneous embedded hardware, including multiple MCU families (e.g., ESP32 and nRF52) as well as emerging RISC-V–based system-on-chips. This study will systematically benchmark classification accuracy, inference latency, memory footprint, and energy consumption under realistic deployment constraints, providing a more general assessment beyond a single hardware platform. Second, although depthwise separable convolutions were adopted in this work to achieve a favorable accuracy–efficiency trade-off, additional hardware-aware optimization techniques will be explored. These include grouped convolutions, mixed-precision inference, and adaptive or fine-grained quantization strategies, aiming to further reduce computational overhead and memory usage on severely resource-constrained devices. Third, the current study relies exclusively on RGB imagery. We acknowledge that incorporating multispectral or temporal information has the potential to significantly improve robustness under varying illumination conditions, soil backgrounds, crop growth stages, and environmental factors. Due to space limitations and the primary objective of assessing the feasibility of TinyML-based weed classification on ultra-low-power devices, such extensions were not included in this work. Nevertheless, we are actively preparing follow-up studies leveraging high-throughput multispectral plant phenotyping platforms, with multimodal data fusion forming a core component of future research. In terms of robustness and generalization, future evaluations will be extended to multiple crop types and farm-scale datasets, in conjunction with diverse embedded platforms. This will enable a more comprehensive analysis of cross-domain generalization beyond a single dataset or hardware configuration. Finally, although TinyWeedNet is designed as a classification model, prior studies have demonstrated the feasibility of deploying image segmentation models on microcontrollers. Building upon this foundation, future work will investigate MCU-compatible weed segmentation and localization models, enabling closed-loop perception–control–actuation pipelines for fully autonomous and energy-efficient weed management systems.

In summary, this study demonstrates that TinyML-oriented lightweight CNNs, when systematically evaluated and optimized using hardware-level measurements, offer a viable pathway toward reliable, energy-efficient, and autonomous weed identification in precision agriculture.

## Data Availability

The raw data supporting the conclusions of this article will be made available by the authors, without undue reservation.

## Footnotes

1
https://stm32ai.st.com/stm32-cube-ai/

2
https://pytorch.org/

3
https://onnx.ai/
